# Supplementary material for: Identifying effective surveillance measures for swine pathogens using contact networks and mathematical modeling
Source: PLoS One. 2025 Aug 22;20(8):e0329714. doi: 10.1371/journal.pone.0329714 (PMC12373290; doi:10.1371/journal.pone.0329714)
Supplement: S1 Tables — Table A: Pig holding and tour characteristics; Table B: PRRS index case weights; and Table C: APP and ASF index case weights. (PDF) [file pone.0329714.s001.pdf]

Supporting Information.
S1 Tables

Year	Total Number of Holdings	Mean Number of Pigs per Holding	Total Number of Direct Transports	Mean Number of Pigs per Direct Transport	Mean Number of Holdings per Tour
2014	12,268	143	184,508	26	5
2015	12,015	145	182,160	26	5
2016	11,759	142	179,878	26	5
2017	11,436	145	178,076	26	5
2018	11,079	147	172,110	26	5
2019	10,671	147	169,483	26	5

Table 1. Pig Holding and Tour Characteristics

Parameters			Weights
**Farm Type**	**Border**	**Outdoor Access**	
Farm without sows	No	1	1
Farm without sows	No	2	2
Farm without sows	No	3	4
Farm without sows	No	4	8
Farm without sows	Yes	1	2
Farm without sows	Yes	2	4
Farm without sows	Yes	3	8
Farm without sows	Yes	4	16
Farm with sows and boars	No	1	2
Farm with sows and boars	No	2	4
Farm with sows and boars	No	3	8
Farm with sows and boars	No	4	16
Farm with sows and boars	Yes	1	4
Farm with sows and boars	Yes	2	8
Farm with sows and boars	Yes	3	16
Farm with sows and boars	Yes	4	32
Farms with sows without boars	No	1	4
Farms with sows without boars	No	2	8
Farms with sows without boars	No	3	16
Farms with sows without boars	No	4	32
Farms with sows without boars	Yes	1	8
Farms with sows without boars	Yes	2	16
Farms with sows without boars	Yes	3	32
Farms with sows without boars	Yes	4	64

Table 2. PRRS Index Case Weights.

Parameters		Weights
**Wild Boar Risk**	**Outdoor Access**	
No	1	1
No	2	2
No	3	4
No	4	8
Yes	1	2
Yes	2	4
Yes	3	8
Yes	4	16

Table 3. APP and ASF Index Case Weights.
