# Supplementary figures and images for: Identifying effective surveillance measures for swine pathogens using contact networks and mathematical modeling
Source: PLoS One. 2025 Aug 22;20(8):e0329714. doi: 10.1371/journal.pone.0329714 (PMC12373290; doi:10.1371/journal.pone.0329714)

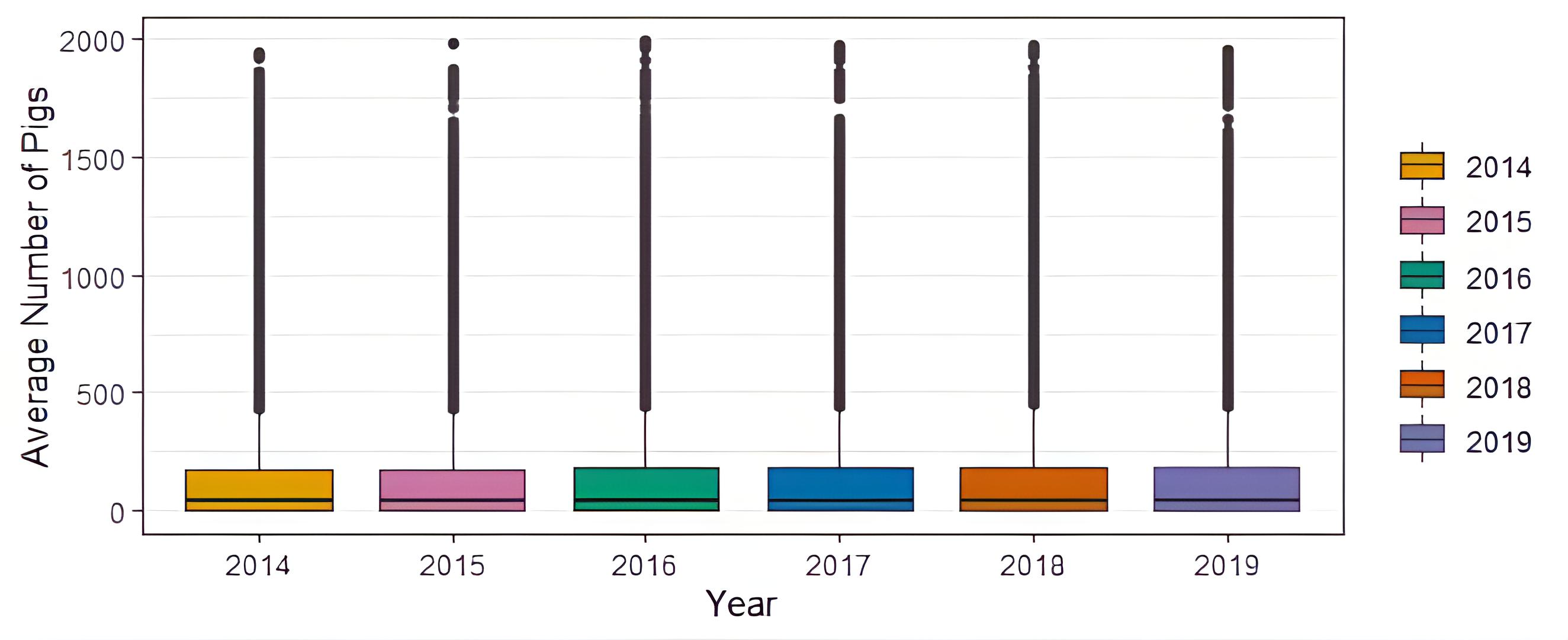

Supplement: S1 Fig — (TIFF) [file pone.0329714.s002.tiff]

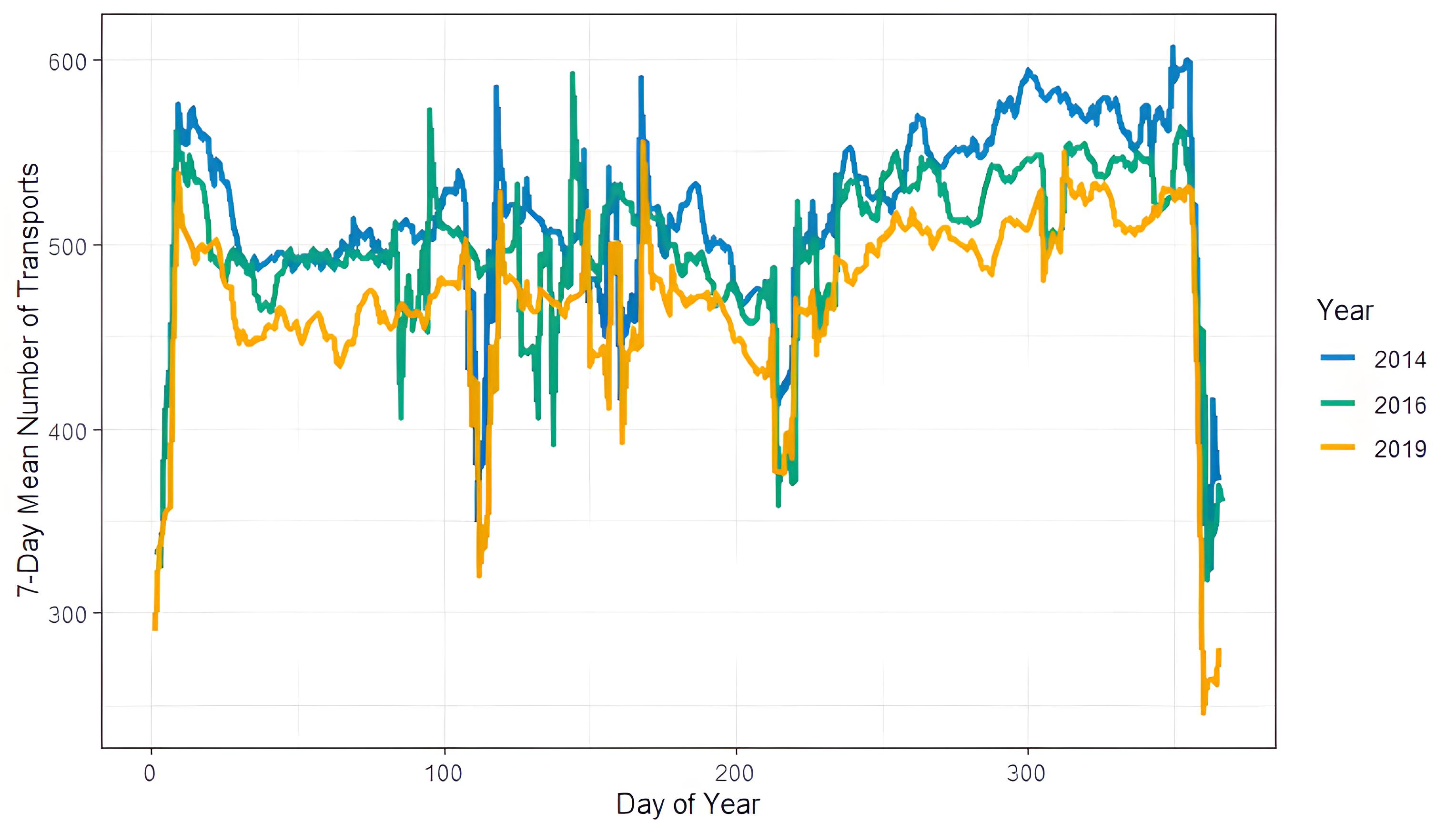

Supplement: S2 Fig — January 1, 2014 through January 6, 2014 are not included. (TIFF) [file pone.0329714.s003.tiff]

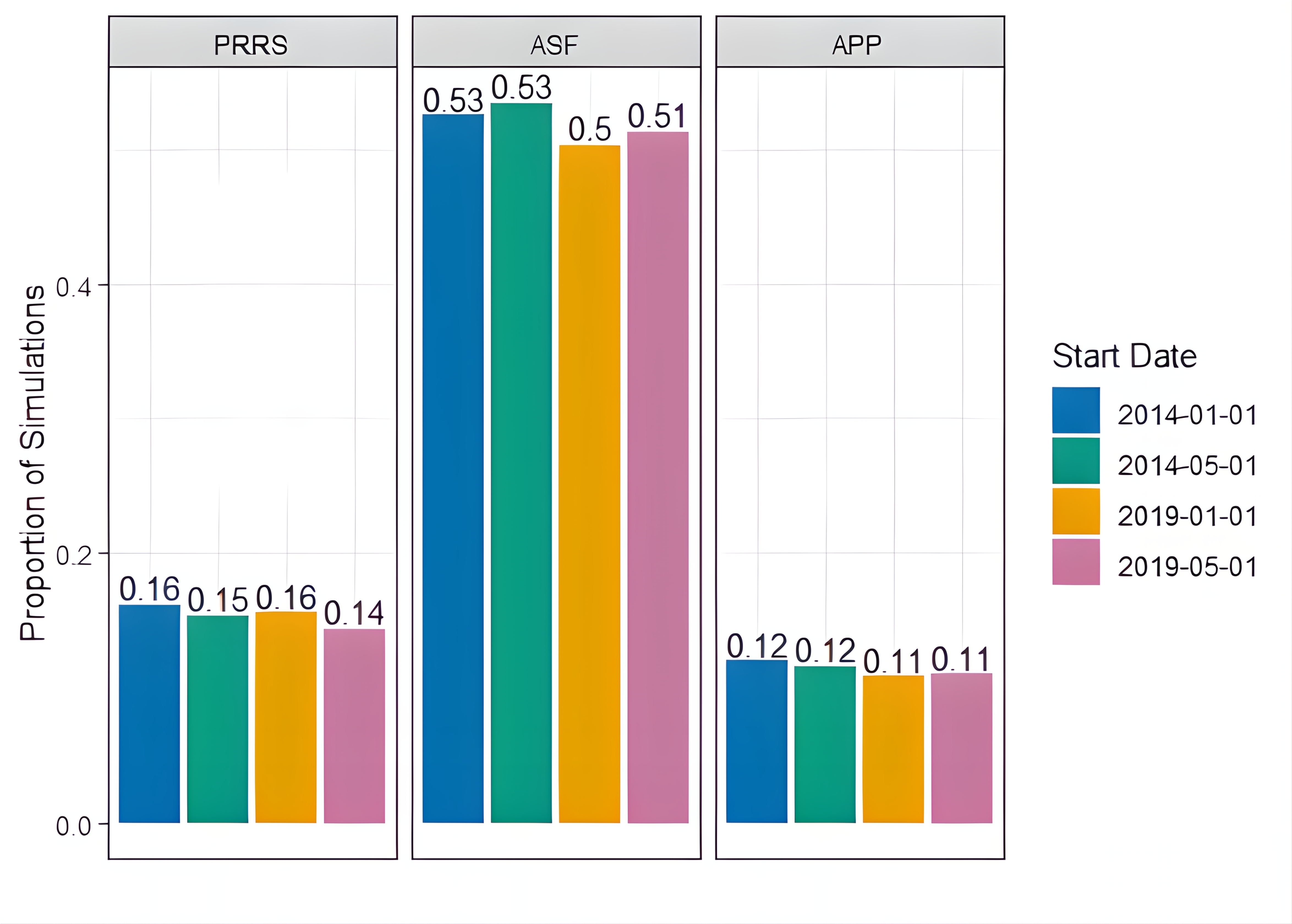

Supplement: S3 Fig — A simulation is classified as having a large outbreak when at least 10 or more farms have been infected. (TIFF) [file pone.0329714.s004.tiff]

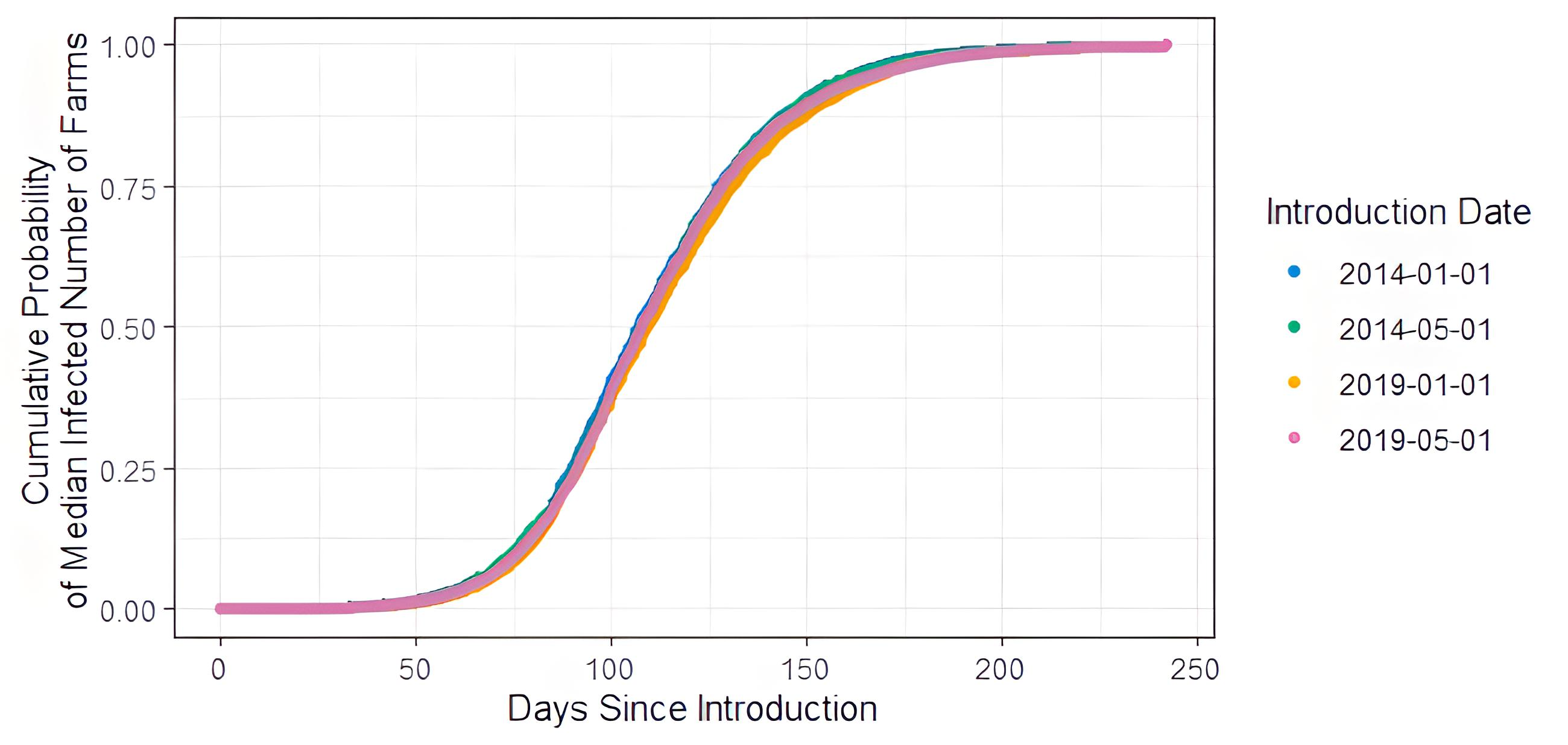

Supplement: S5 Fig — (TIFF) [file pone.0329714.s006.tiff]

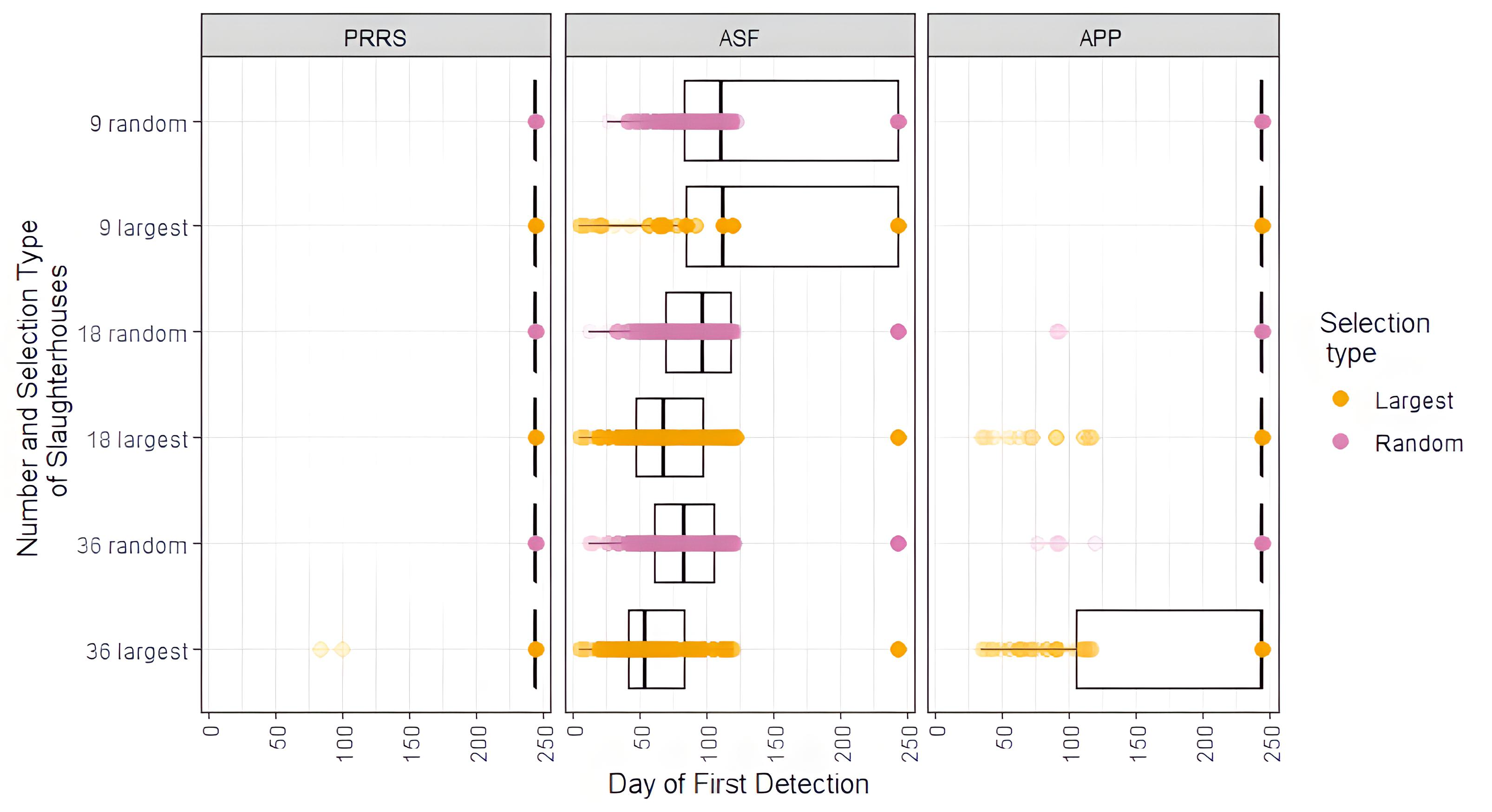

Supplement: S6 Fig — Each circle represents the first date a positive case was detected for the simulation run. Only simulations that lead to large outbreaks at the end of the 8 months simulation period considering no control measures are included. (TIFF) [file pone.0329714.s007.tiff]

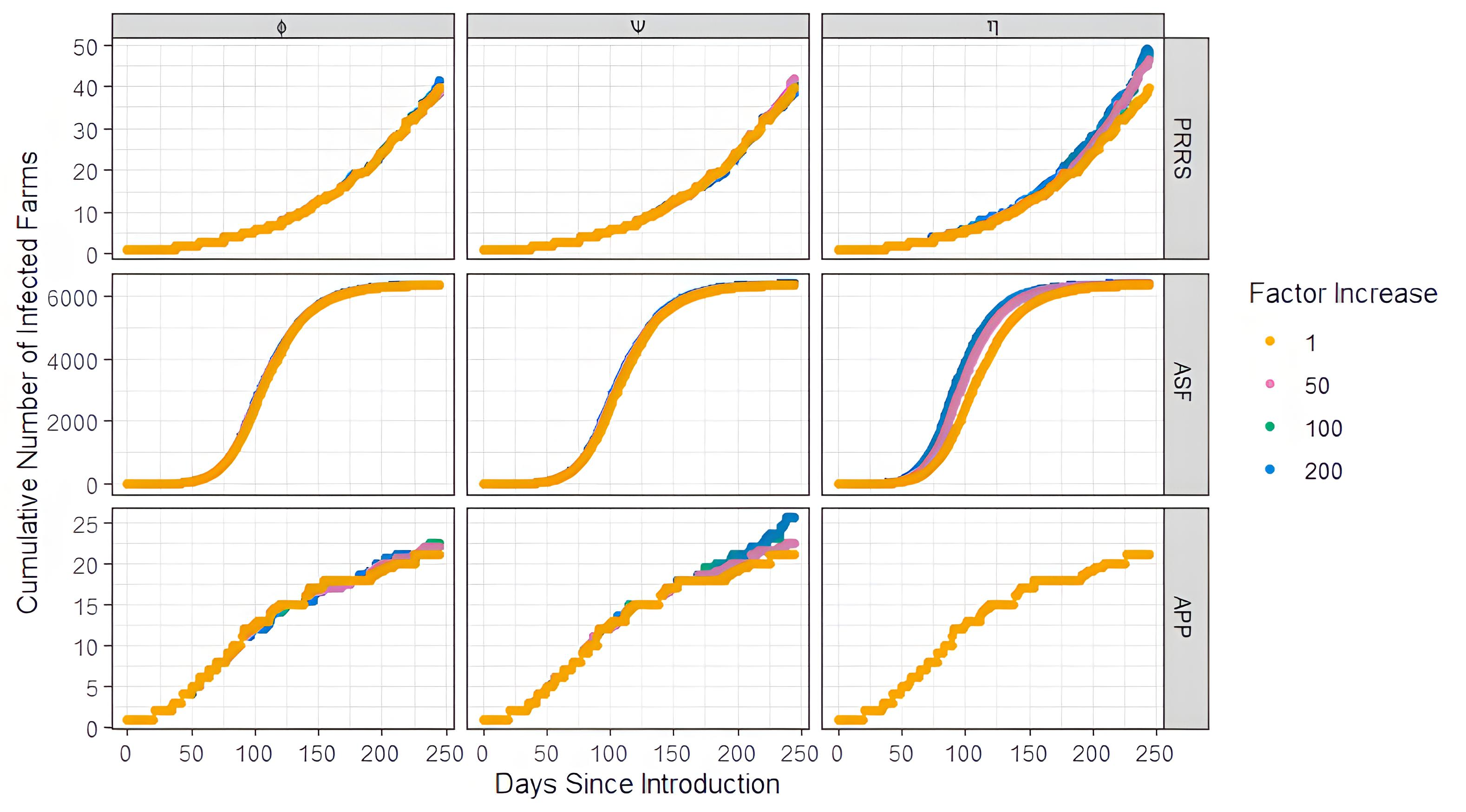

Supplement: S7 Fig — ϕ is the direct truck share transmission parameter, ψ is indirect truck share transmission parameter, and η is exterior truck and truck driver transmission parameter. Results are from a disease introduction from May, 2019 and from simulations with large outbreaks only. We plot only the baseline and factors beginning at 50 because smaller values resulted in minimal change in cumulative number of infected farms. (TIFF) [file pone.0329714.s008.tiff]

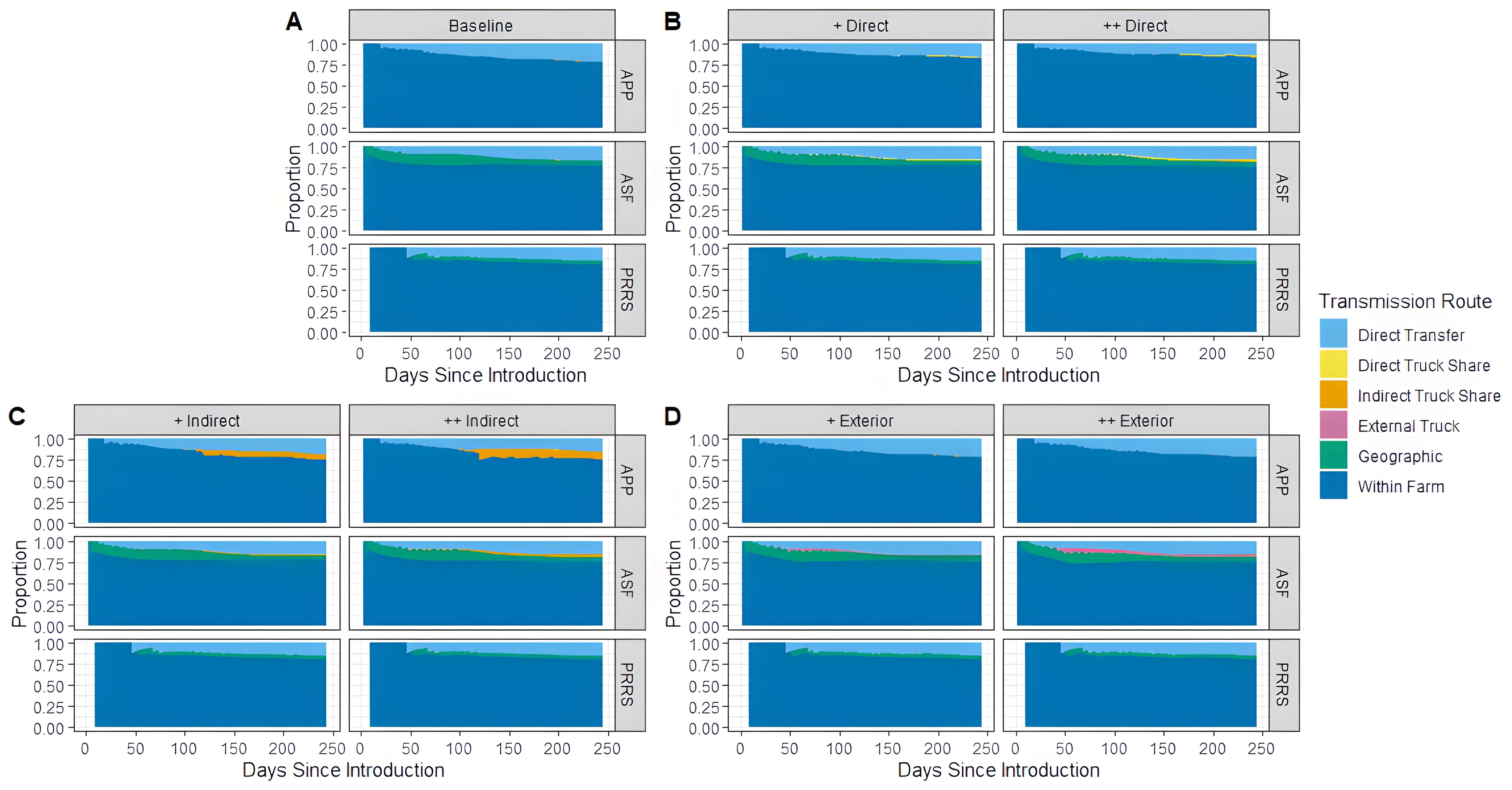

Supplement: S8 Fig — (A) Baseline parameters (B) An increase of the direct truck share parameter (ϕ) by a factor of 50 (+ Direct) and 100 (++ Direct) (C) An increase of indirect truck share parameter (ψ) by a factor of 50 (+ Indirect) and 100 (++ Indirect) (D) An increase of the exterior truck fomite parameter by a factor of 50 (+ Exterior) and 100 (++ Exterior). (TIFF) [file pone.0329714.s009.tiff]

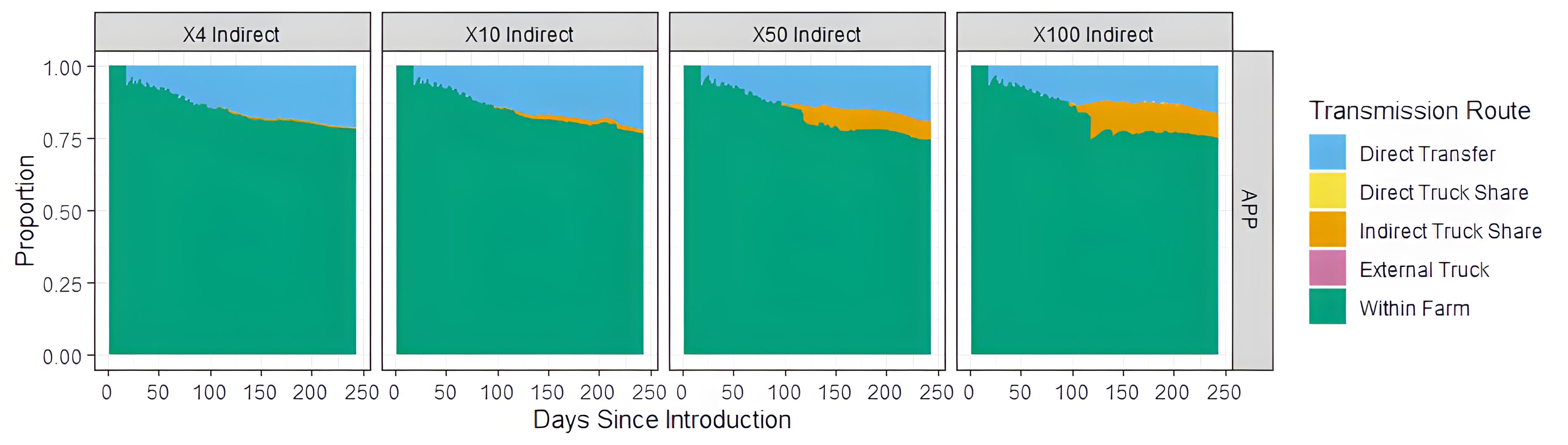

Supplement: S9 Fig — (TIFF) [file pone.0329714.s010.tiff]

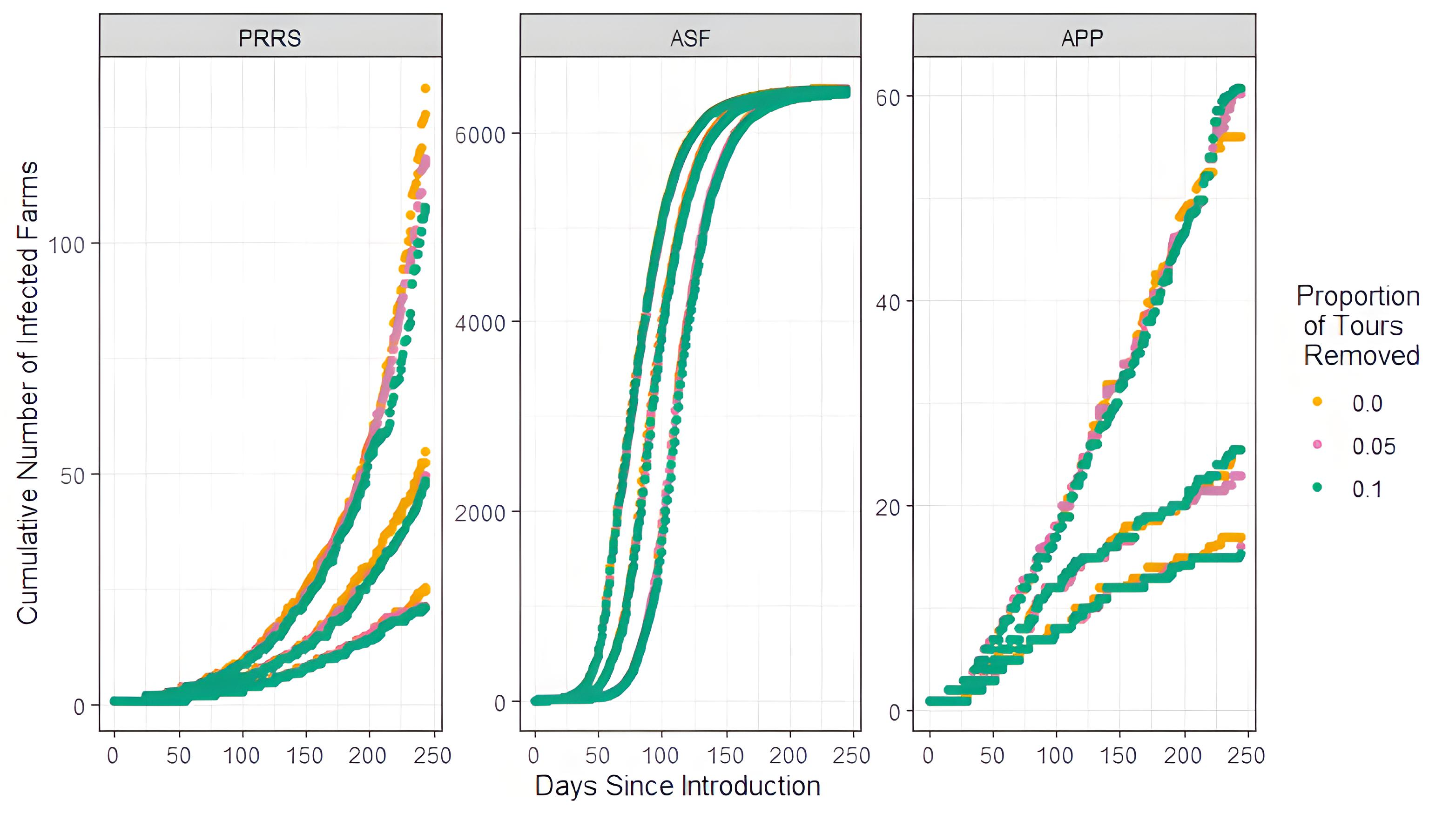

Supplement: S10 Fig — Only simulations with large outbreaks and with a disease introduction in May 2019 are included. (TIFF) [file pone.0329714.s011.tiff]

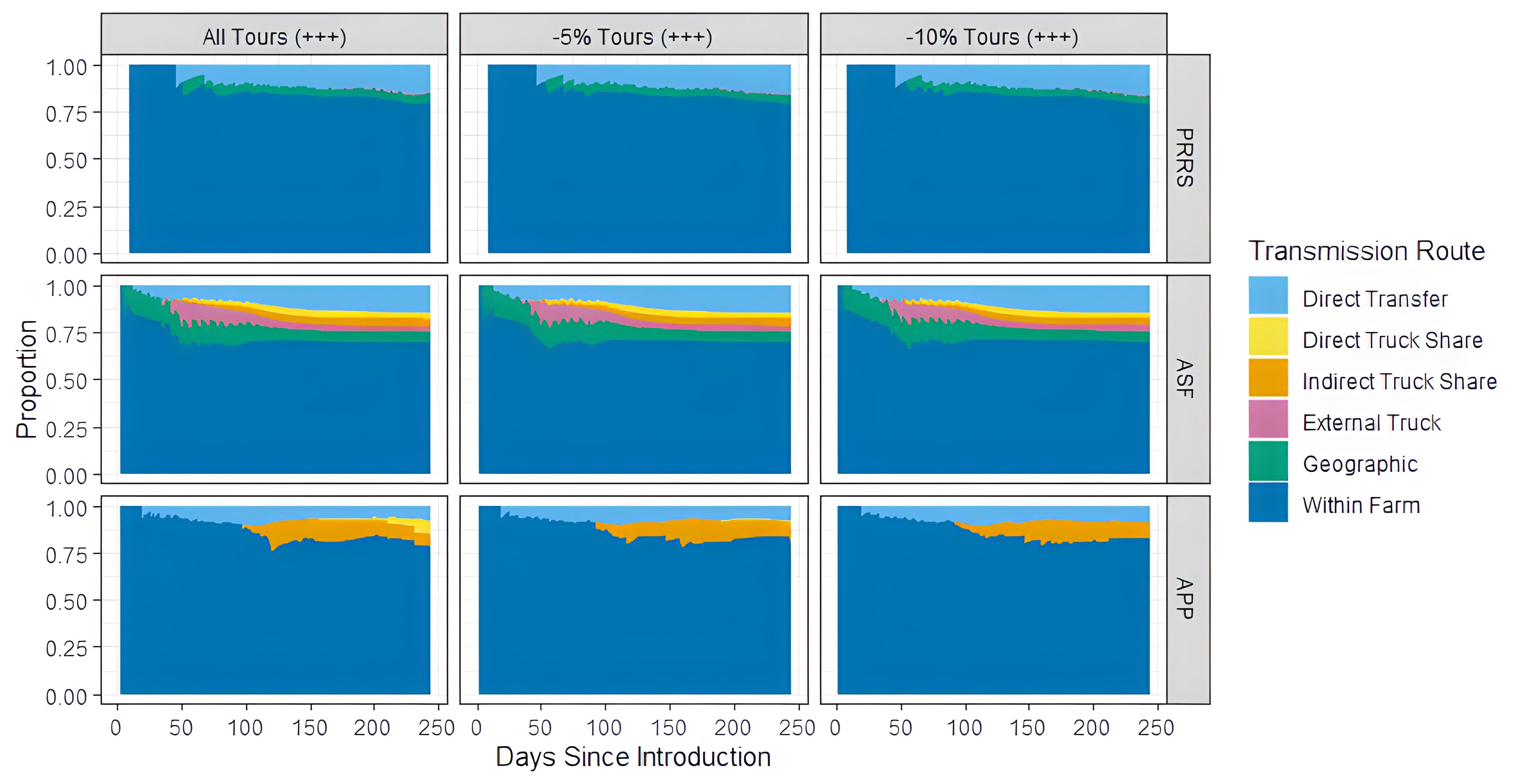

Supplement: S11 Fig — All Tours indicates the results with all of our current tour data, -5% are results after a random selection of 5% of tours are removed, -10% is the same but for 10%. (+++) indicates that the tour parameters are increased by a factor of 200. The model results are only simulations with large outbreaks and with a disease introduction in May 2019. (TIFF) [file pone.0329714.s012.tiff]

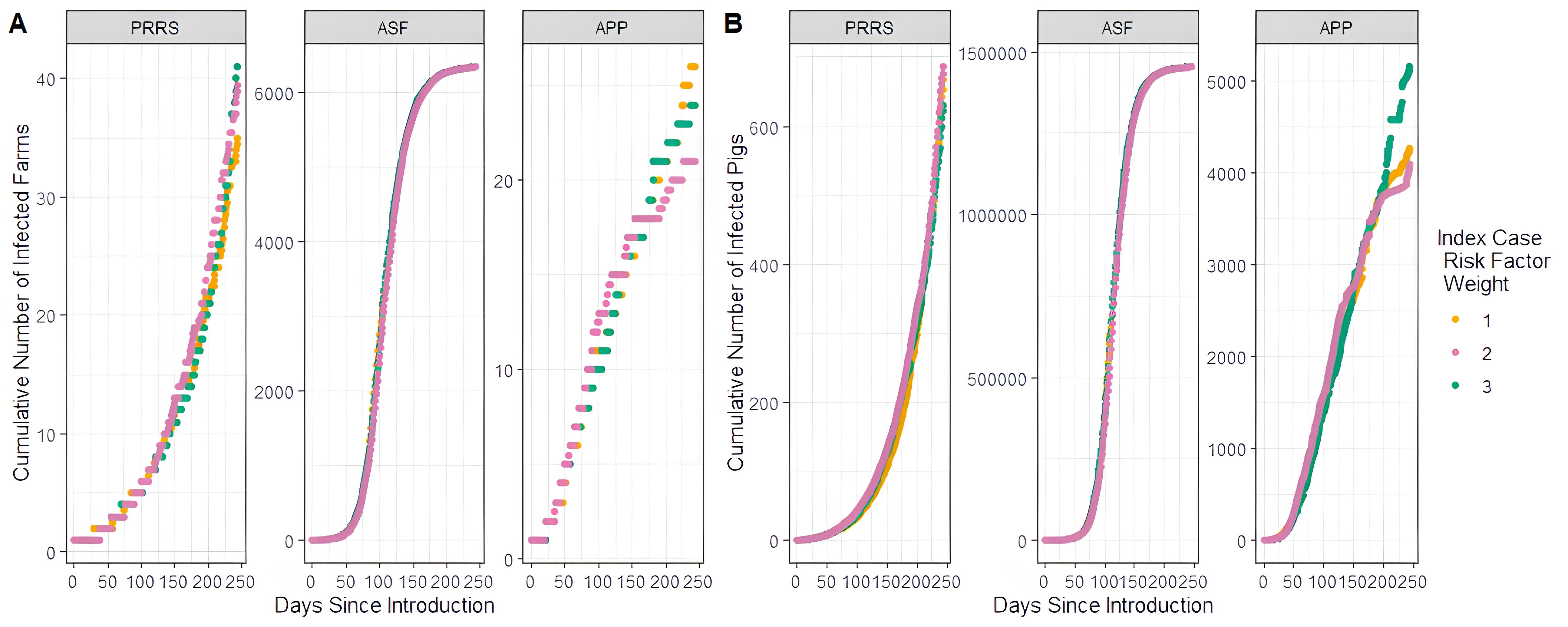

Supplement: S12 Fig — Median cumulative number of infected farms (A) and pigs (B) for each disease with different index weight schemes. Index case weight factor 1, is the model run with uniform random selection of farms while factor 3 is the model run where each increase level of risk for the farm, increases the weight of index case selection by a factor of 3. No surveillance and an introduction date in May 2019 were used. (TIFF) [file pone.0329714.s013.tiff]
