# Supplementary material for: Identifying effective surveillance measures for swine pathogens using contact networks and mathematical modeling
Source: PLoS One. 2025 Aug 22;20(8):e0329714. doi: 10.1371/journal.pone.0329714 (PMC12373290; doi:10.1371/journal.pone.0329714)
Supplement: S1 Appendix — (PDF) [file pone.0329714.s014.pdf]

Supporting Information.
S1 Appendix

Section A. Sensitivity analysis tour data After removing 5% and 10% of the tours and increasing the tour parameters by a factor of 200, we simulated 1,000 runs of the model and calculated the daily median cumulative number of infected farms along with the first and third quartiles. The results are shown in S10 Fig. On the last day of the model run (Days Since Introduction = 244) for ASF, there was a 0.2% increase of median cumulative infected farms when comparing the 10% reduced tour data with the full tour data. For APP, there was 0% change. For PRRS, there was an increase of 7 infected farms, a 13% increase in the median cumulative infected number of farms. To test if the difference between the number of infected farms for 10% and 0% tour reduction for PRRS was statistically significant, we performed a Mann-Whitney U-test on the distribution of infected number of farms at day 244. The two-sided p-value was 0.3253, indicating no evidence of a difference between the number of infected farm distributions.

We also calculated the proportion of cases transmitted via each pathway with the 5% and 10% of tours removed. The results are shown in S11 Fig. For PRRS and ASF, there is no change in proportion of cases from the full tour data. For APP, after 200 days since disease introduction, there was a change in proportion of direct truck share cases. At 244 days, when 10% of tours were removed and tour parameters were increased by 200, 0% of new cases were via direct truck sharing. If we add back the tour data that we removed, then the percent of direct truck share increases to 7.5%. No other transmission pathway increases by more than 1%. This could be because APP has a higher direct truck share transmission rate and is therefore more susceptible to increases of direct truck share contacts. It is important to note that the 7.5% of direct truck share transmission for APP with all tour data is only observed after the tour parameters have increased by a factor of 200.

Section B. Sensitivity analysis of index case The index case weighting algorithm implemented to increase a farm's probability of selection as an index case was based on the farm's risk level explained in Section 2.6. For each increase in risk level, the index case weight increased by a factor of 2. Because 2 was chosen arbitrarily, we ran the model with factors 1 (so there is no weighting of index cases) and 3 to analyze the sensitivity of the factor to model outcomes. The differences in the outcomes were negligible. (See S12 Fig for the results.)
