# Supplementary material for: Identifying effective surveillance measures for swine pathogens using contact networks and mathematical modeling
Source: PLoS One. 2025 Aug 22;20(8):e0329714. doi: 10.1371/journal.pone.0329714 (PMC12373290; doi:10.1371/journal.pone.0329714)
Supplement: Attachment — Submitted filename: Response to Reviewers.pdf [file pone.0329714.s017.pdf]

Swiss TPH
Swiss Tropical and Public Health Institute

Kathleen Moriarty
kathleen.moriarty@swisstph.ch
+41 76 799 44 67

Disease Modelling Unit
Epidemiology and Public Health
Swiss Tropical and Public Health Institute
Kreuzstrasse 2
4123 Allschwil
Switzerland

PLOS One

April 30, 2025

Dear Editors,

We thank the Academic Editor and Reviewers for their constructive feedback on our manuscript titled “Identifying effective surveillance measures for swine pathogens using contact networks and mathematical modeling.” We are grateful for the recognition of the relevance, novelty, and methodological rigor of our study, and we have carefully considered each comment.

In the following pages, we provide a detailed, point-by-point response in red font embedded directly within the reviewers’ comments. Where relevant, we include revised manuscript text and additional explanations.

We hope these revisions and explanations sufficiently address the concerns raised and improve the clarity and impact of our work.

We appreciate the opportunity to revise our manuscript.

Kind regards,

Kathleen Moriarty, Nakul Chitnis, Salome Dürr, Antoine Champetier, Francesco Galli

Reviewer #1

General Comments

1. Relevance and Significance

- The manuscript explores a crucial topic, examining the spread of swine pathogens through multiple transmission pathways including direct transport, truck tours, and local spread and evaluating the effectiveness of different surveillance strategies in timely pathogen detection.
- The comparative approach using PRRS, ASF, and APP which have distinct biological and epidemiological profiles is particularly valuable. It highlights how differences in transmission modes affect the dynamics and detection of disease in pig populations.

2. Novelty

- The integration of three data sources (TVD records, pig trader data, and AGIS holdings data) to construct a complex, multi-layered contact network is a strong point of the study.

· Addressing both endemic (APP, PRRS) and emerging threats (ASF) in one modeling framework is quite innovative and provides a broader epidemiological scope than single-disease models.

3. Methodological Approach

· Overall, the approach (SEIAR within-herd model + dynamic, labeled, between-farm contact network) is logical and seems to capture key processes of disease transmission.

· The differentiation among direct transport, indirect truck-sharing, and fomites via truck drivers/equipment is valuable but still relatively underexplored in the literature. The authors make a genuine effort to incorporate real-world heterogeneities where data allow.

4. Clarity and Organization

· The manuscript is generally well-organized. The distinct sections (Introduction, Methods, Results, Discussion) are clearly delineated.

· Some sections, especially the Results, occasionally become quite dense when describing multiple pathogens and numerous surveillance scenarios. Additional structuring of the results possibly using subsections with descriptive headings would improve readability.

We acknowledge that the Results section covers a range of analyses across the different pathogens and surveillance scenarios, which has led to a high level of detail. To improve the clarity and readability, we had already structured the Results section into 5 subsections: (1) Seasonality and annual trends, (2) Routes of transmission, (3) Surveillance, (4) Sensitivity analysis of tour parameters ϕ, ψ and η and tour data, and (5) Sensitivity analysis of index case. We prefer this to the alternative of organising the Results by disease because it allows us to focus on aspects of transmission and control across diseases rather than focus on particular diseases.

5. Limitations

· The study acknowledges data gaps on truck tours (only 15% coverage), which constrains definitive conclusions on indirect and fomite-based transmission. While the sensitivity analyses mitigate these concerns somewhat, the limitations should be emphasized more.

We agree that the limited availability of the full truck tour data is a limitation in our model. We have emphasized this in the Section 2.2 Sources of Data, Section 2.10 Sensitivity analysis and the Discussion. A more detailed response addressing this point is included in our reply to the relevant Section-by-Section Comment below.

· The homogeneous mixing assumption at the farm level simplifies within-herd dynamics. Further consideration of farm size and housing system might affect the results, especially for highly transmissible pathogens (ASF) versus those with more variable clinical presentations (APP, PRRS).

We acknowledge that the homogeneous mixing assumption can impact the model outcomes. We have expanded our justification for using homogeneous mixing with frequency-dependent transmission, particularly within the Swiss context where farm density is regulated by law, in Section 2.3 Within-Farm Layer. A more detailed explanation is provided in our response to the relevant Section-by-Section Comment below.

6. Impact

- The findings on how quickly ASF can spread underscore the urgency for robust surveillance measures.
- The conclusions that farmer-based surveillance detects outbreaks earlier than other strategies has direct policy relevance particularly because it aligns with recent real-world experiences of ASF spread in Europe.
- The large potential for ASF spread in a naive country emphasizes the need for preparedness, which this model can inform.

Section-by-Section Comments

Abstract

1. The Abstract clearly summarizes the key objectives (assessment of contact types, evaluation of surveillance strategies).

Thank you for this comment.

2. You might emphasize the key quantitative finding: "Direct truck transport and local spread were found to be the main routes …" This is a strong conclusion that guides the reader upfront.

We have revised this statement to use active voice and added further emphasis. Updated text: *Our findings highlight that direct truck transport and local spread are the main routes of between-farm transmission.*

3. Consider briefly mentioning the major takeaway on surveillance (farmer-based strategies were the most effective).

The major takeaway regarding surveillance, farmer-based strategies being the most effective, is already stated in the abstract, where we highlight the quantitative differences. Specifically, *farmer-based surveillance programs were the only measures that consistently identified outbreaks of APP and PRRS, and they detected ASF outbreaks almost 8 weeks earlier than active slaughterhouse- and network-based surveillance.*

Author Summary

1. The lay summary is concise and effectively highlights the real-world relevance.

Thank you for this comment.

2. The statement “It is not clear which contact type has the highest probability of transmission...” is appropriately answered in the subsequent text, but you might consider a more direct statement of your findings there.

We have updated the text to highlight the knowledge gap to lead into our model description. Updated text states: ***However, the relative contribution of these different contact types to disease spread remains unclear. Likewise, the effectiveness of existing and proposed surveillance programs is uncertain.*** *Once an outbreak begins to spread, detecting it quickly is vital. We built a model based on data from Swiss pig holdings and transports. We simulated the spread of three infectious pathogens within and between farms, based on their different types of contact. The model simulations showed that the direct transport of pigs and geographic proximity were the main pathways of between-farm spread. We also found that surveillance programs based on farmer reporting were the most effective.*

Introduction

1. The motivation for comparing ASF, PRRS, and APP is clear, but you may want to highlight more systematically why these three were chosen as representative of distinct modes of transmission or clinical severity.

We have expanded the sentence which introduces these diseases to include: `` — which represent distinct transmission characteristics and endemicity levels”. . The rationale for selecting ASF, PRRS, and APP, and their distinct modes of transmission and clinical severity, is further discussed in detail in Section 2.1 of the Materials and Methods. Additionally, Table 1 provides a side-by-side comparison of these pathogens.

2. The reference to historical data (Figure 1) is helpful to show the Swiss context. Ensuring that the figure is easy to read and interpret is important. You could consider highlighting regions with prior cases more visibly (e.g., color-coding or labeling hotspots).

We have already color-coded the regions in Figure 1 to highlight areas with prior cases. We cannot explicitly label areas with prior cases due privacy issues.

3. The hypotheses are generally well-articulated, but you might specify if you hypothesized that direct pig transports would be the most critical path or if you expected major differences in detection times among the three pathogens.

We expected the mode of transmission of the pathogen to have an impact on the performance of the surveillance strategies, and correspondingly the detection time, as stated in the following sentence in the Introduction: *We hypothesize that pathogens with distinct transmission dynamics result in different performance of surveillance measures.* However, we did not a priori specifically hypothesize that direct pig transports would be the most critical pathway for all pathogens.

Materials and Methods

1. Data Sources (Section 2.2)

- The coverage of TVD, trader data, and AGIS is appropriate. The detail about not having the full sequence of truck stops for all transports is a key limitation. The authors do note this, but it merits repeated emphasis, given its impact on certain contacts (tour-based).

We state in the data description that “Since these two trader companies do not service all farms in Switzerland, we do not have the truck number, collection time, nor delivery time for all records of the TVD data.” We provide the exact coverage of 15%. All of section 2.10 is related to the shortcomings of the missing 85% and the sensitivity analysis that we had done. We also discuss this shortcoming in the Discussion section with an entire paragraph, starting with:
Due to the limited published estimates on transport tour transmission rates of direct and indirect truck sharing and truck fomites, setting the parameter values for our model was difficult…

- It is beneficial that you combine farm attributes (farm type, pig numbers) with geographical coordinates. Clarify whether any missing data were excluded, or if imputation was performed for incomplete records.

For a limited subset of farms where precise geographic coordinates were not available, we used the centroid of the municipality's polygon as an approximation of the farm location. This imputation allowed us to retain these farms in the analysis while maintaining a reasonable approximation of their location. We have updated the manuscript in Section 2.2 Sources of Data with this additional text: *For a limited subset of farms where precise geographic coordinates were not available, we used the centroid of the municipality's polygon as an approximation of the farm location. This allowed us to retain these farms in the analysis while maintaining a reasonable approximation of their location.*

2. Within-Herd Model (SEIAR) (Section 2.3)

- The compartmental structure is clearly described.

Thank you for this comment.

- The homogeneous mixing assumption is common but can lead to overestimation of outbreak size in large farms. A brief justification is provided; perhaps highlight any known empirical evidence that suggests frequency dependence within typical Swiss pig farms.

We agree that the choice between frequency- and density-dependent transmission can affect model outcomes, especially in the context of large farm sizes. As you suggest, we have expanded our justification to clarify why frequency-dependent transmission is appropriate in the context of Swiss pig farms in Section 2.3 Within-Farm Layer. Updated text: *In addition, we assumed homogeneous mixing of pigs at the farm with frequency dependent transmission.*

Although density-dependent transmission has often been used for animal diseases, this is more appropriate for animals kept in open spaces such on pasture where densities can change. Pig farms in Switzerland are strictly regulated with a clearly defined maximum number of animals per space by legislation. This leads to a maximum number of pigs per barn, limiting the number of contacts for each animal, even if the number of pigs per farm increases. Hence a frequency-dependent transmission assumption is more appropriate across farm systems in Switzerland.

Original text: *In addition, we assumed homogeneous mixing of pigs at the farm with frequency dependent transmission. Although density-dependent transmission has often been used for animal diseases, this is more appropriate for free-roaming animals in wide open spaces or those owned by pastoralists. Pig farms in Switzerland are more strictly regulated with larger farms having more space, limiting the number of contacts, as the number of pigs increases. Hence a frequency-dependent transmission assumption is more appropriate across farm sizes in Switzerland.*

3. Between-Farm Contact Networks (Section 2.4)

- The categorization of edges (D, P, I, T, G) is thorough. The distinction among direct pig transport, shared truck contacts (direct/indirect), and truck fomites is valuable.

Thank you for this comment.

- For geographic edges, clarify why you selected a specific threshold \(k \) for distance (e.g., 5 km, 10 km). A sensitivity test for different distance cutoffs might strengthen the argument that “local spread” remains important even if the radius changes slightly.

Thank you for the suggestion to clarify the choice of the distance threshold **k**. We selected 2 km as the cutoff based on the literature from which we derived our geographic spread parameters. Specifically, the publication we referenced used a 2 km threshold for local spread, and we adopted this same value for consistency with prior studies.

We agree that a sensitivity analysis for different distance cutoffs could further strengthen our argument regarding the importance of local spread. However, given the scope of our study, we did not conduct a sensitivity test for varying distance thresholds. We have updated the manuscript, particularly the "Between-herd transmission" paragraph in the Parameters section, to better clarify the reasoning behind the 2 km threshold. Here is the additional text:

To be consistent with the Stegeman et al. (2002) study, we chose a geographic spread threshold (k) as described in Section 2.5 to be 2 kilometers.

4. Model Implementation and Parameters

· More detail on how the authors set the parameters for PRRS, ASF, and APP (especially for indirect truck sharing probabilities) would be useful. The references for these parameter estimates are critical for the credibility of the model.

The parameters used for PRRS, ASF, and APP are provided in **Table 4** (within-farm parameters) and **Table 6** (between-farm parameters), with detailed explanations in Section 2.7 (Parameters section).

Additionally, we detailed the estimates here: *For the indirect truck-sharing parameters (ψ), the pathway of transmission is predominantly through urine and feces. Several studies have concluded that nasal and rectal secretions are not significant transmission drivers for either CSFv or ASFv (Dewulf et al., 2002; Pietschmann et al., 2015). For ASF, we use the same ratio of ψ to ϕ as for CSF with evidence of ASF transmission through indirect contact in recent outbreaks in Estonia (Nurmoja et al., 2020). PRRSv has been shown to spread easily through urine, nasal, and feces secretion and through truck fomites (Dee et al., 2004a; Otake et al., 2002). We therefore chose to increase the ratio of indirect truck-sharing rate, ψ, to ϕ for PRSS as compared to the ratio of ψ to ϕ for CSF to account for this increased rate of transmission. In the absence of concrete data on the relative ratio of ψ to ϕ for PRRS, we make arbitrary choice of using 1.5 as the inflation factor. In Section 2, we conduct additional sensitivity analysis to consider the impact of this choice.*

· In the sensitivity analysis, the authors do a robust job exploring large multiplicative changes in tour-related parameters. This is well-executed but might benefit from additional clarity on how these factors relate to realistic upper/lower bounds from literature.

In the sensitivity analysis, we chose the upper and lower bounds for the tour-related parameters based on the order of magnitude of realistic variations in these factors, rather than directly deriving them from the literature, since there is unfortunately little literature on these parameter values. Our approach was to explore a range of values that we believed could realistically influence the outcomes, stopping when we began to observe notable changes in the results. While these bounds were not strictly defined by literature, we aimed to cover a sufficient range of possible scenarios to assess the robustness of our findings.

5. Surveillance Measures

· The wide range of surveillance approaches (farmer-based with different participation and thresholds, slaughterhouse-based, targeted network-based) is helpful in illustrating detection trade-offs.

Thank you for this comment.

- The frequency of sampling (every 90 days for targeted network-based) is an important assumption. Justify briefly why 90 days was chosen as opposed to, say, 60 or 120 days.

We chose 90 days as the most practical approach. The 90 days were chosen to balance between a realistic and useful approach for farm-based surveillance. Breeding farms are visited four times per year by private pig health services, fattening farms once a year. These farm visits can be used to collect data for such a surveillance programme. More often than every 90 days is thus not realistic in terms of resources used. Therefore we chose the highest realistic frequency for the Swiss pig health service system.

Additional text added to the manuscript in section 2.8 Surveillance Programs and Control Measures: *The 90 days were chosen to balance between a realistic and useful approach for farm-based surveillance, adapted to the visiting schedule of pig health services in Switzerland.*

Results

1. Overall Disease Spread

- The use of median, 95th percentile, and maximum outbreak sizes is appropriate, but it is easy for the reader to lose track of which pathogens are being referred to. Consider structuring the text so each pathogen is discussed in a separate subsection (or create tables/visual aids with side-by-side results).

We have already structured our visuals to display side-by-side results for each pathogen. Please refer to Figure 5, Figure 6, Figure 7, and so on, where the results for each pathogen are presented in parallel. We hope these figures help clarify the comparison across pathogens.

Highlighting that only 16% of PRRS and 11% of APP simulations resulted in large outbreaks is an important detail. Possibly include a short explanation regarding whether that low probability is consistent with their known epidemiology in Switzerland.

In the Results section, we have added the following text*: Across all simulations, only 16% of PRRS runs and 11% of APP runs led to large outbreaks, compared to half of the simulations for ASF.*

This observation is presented without interpretation of the Swiss epidemiology, as we intended to save that context for the Discussion section.

In the Discussion section, we already had this text: *Unfortunately, we could not validate our model to disease data since positive cases of PRRS and APP are very rare in Switzerland, and ASF has yet to reach it. These few cases are reflected in our model predictions with only 16% of PRRS and 11% of APP simulations resulting in outbreaks.*

2. Seasonality and Annual Trends

· While you conclude no statistically significant differences in outbreak trajectories by introduction date, it might be helpful to present these results in simpler figures (boxplots or smaller sub-panels) for each pathogen. This would complement the discussion about the minor role of seasonality in Switzerland.

We had already included plots for each pathogen, which can be found in Figure 6. These plots visually represent the outbreak trajectories by introduction date and are intended to complement the discussion on the relatively minor role of seasonality in the Swiss context.

· You reference a Kolmogorov–Smirnov test. Consider whether an additional measure like effect size or visual distribution plots might clarify these comparisons.

We had already included visual cumulative distribution plots for ASF, which can be found in **Supplementary Section, Figure 5**. These plots were included to provide additional context and clarity, helping to further illustrate the results from the Kolmogorov–Smirnov test.

3. Routes of Transmission

· The percentage breakdown of between-farm spread is a key output. Showing direct pig transfers and local spread as major drivers is a critical conclusion. The minimal role of tours (<1% on average) is surprising yet informative. Ensure you clarify that these results hinge on partial data for tours. Emphasize that the sensitivity analyses indicate that large multiplication of tour parameters does not drastically change the outcome (except a slight bump for APP).

Added the following text to emphasize this:

Among the between-farm pathways, tour contacts accounted for less than 1% of new cases, regardless of disease. However, it is important to note that the tour contact data were incomplete and may underestimate the true role of this pathway. To address this uncertainty, we conducted sensitivity analyses that scaled up tour-related parameters. These analyses showed that even with significant multiplication of tour frequency and associated risk parameters, the overall contribution of tour transmission remained low.

4. Surveillance Findings

· The detection-time comparisons are crucial for policy. The results, especially regarding the early detection by farmer-based strategies for ASF, are compelling. It would help to provide a single table summarizing average detection time (in days) and the average cumulative infected farms/pigs across all surveillance strategies for each pathogen.

We appreciate the reviewer's comment on the importance of detection-time comparisons for policy. We provided a visual of the earliest detection times for each surveillance program and pathogen for each simulation run in Figure 8. In our results and discussion,

we have emphasized comparisons across pathogens and surveillance measures, rather than focusing on the exact date of detection, as the data is derived from a simulation model. While we understand the potential value of including a table summarizing these details, we believe that the key takeaway from our study lies in comparing the relative effectiveness of the surveillance strategies, rather than pinpointing precise detection dates.

- The differences in performance between slaughter-based and farmer-based strategies could be contextualized in an overall cost or feasibility discussion (though a formal cost–benefit analysis is suggested for future work).

While we agree that the cost and feasibility of different surveillance strategies are important considerations, we felt that conducting a formal cost-benefit analysis was beyond the scope of the current study. Our focus was primarily on comparing the effectiveness of different surveillance strategies in detecting outbreaks. However, we do recognize that a future study could certainly benefit from incorporating such an analysis to provide a more comprehensive understanding of the trade-offs involved in these surveillance approaches.

Discussion

1. Interpretation of Main Findings

- The discussion does a good job contextualizing the results in the broader literature. The strong conclusions regarding the dominance of direct pig transport and local spread, as well as the effectiveness of farmer-based surveillance, are well-articulated.

Thank you for this comment.

- The authors might consider discussing whether geographic spread includes airborne transmission or primarily anthropogenic factors (veterinary visits, shared equipment, etc.). Clarify if future work could stratify or parse those different local routes.

We agree that considering geographic spread, including airborne transmission versus anthropogenic factors, would provide valuable insights into the dynamics of disease transmission. However, given the already extensive scope of our discussion section, we decided to focus primarily on the major aspects of the model and its current limitations. We appreciate this suggestion and will consider it as a direction for future work

2. Model Limitations and Future Directions

- The limitations (partial truck tour data, homogeneous mixing, frequency dependence) are addressed, but consider emphasizing that real-world data on contact intensity, farm biosecurity, and pig flow patterns might refine these estimates.

We have built our model using real-world data on pig flows, which provides a solid basis for understanding between-farm transmission dynamics. We acknowledge that there are limitations due to the lack of comprehensive data on contact intensity, which we address by discussing homogeneous mixing and frequency dependence. Regarding farm biosecurity, we have expanded our discussion to include farm-level biosecurity measures that would add value. We have updated the following text to reflect these considerations:

Nevertheless, the assumptions applied in our model were suitable for studying between-herd transmission. A more detailed within-farm model would require better data on transmission, asymptomatic, and recovery rates of individual pigs, ***as well as more specific farm-level biosecurity measures,*** *which was beyond the scope of our study.*

· Future directions such as including wild boar transmission for ASF, or incorporating more realistic within-farm population structures, are important. Expanding the discussion on how these improvements might change the key outcomes (especially for ASF) would be helpful.

We have added the following final sentence*: These improvements could refine the accuracy of model trajectories by incorporating more detailed information on disease reservoirs, transmission patterns, and the effectiveness of control measures.*

3. Policy Implications

· The conclusion that farmer-based surveillance detects outbreaks faster is consistent with the recently observed ASF outbreaks in Europe and with EFSA's conclusions. Emphasizing how to encourage or mandate better on-farm reporting could strengthen the policy relevance.

While we agree that encouraging or mandating better on-farm reporting is an important aspect of enhancing surveillance programs, we felt that this was outside the scope of our current study. Our focus was on evaluating the effectiveness of different surveillance strategies. However, we recognize the importance of this issue and agree that addressing how to improve on-farm reporting could further strengthen policy recommendations in future work.

· The manuscript might benefit from additional commentary on how these findings could be translated into Swiss or EU-level regulations to enhance early disease detection.

While we agree that translating these findings into Swiss or EU-level regulations to enhance early disease detection is important, this topic was not the primary focus of our study. Our main objective was to evaluate the effectiveness of different surveillance strategies, and as such, we have not delved into specific policy recommendations. That being said, we acknowledge the value of such discussions and agree that future work could explore how these findings could inform regulatory decisions at the national and regional levels.

4. Conclusions

· The final paragraph or “Conclusion” section effectively reiterates the main take-home messages. The recommendation to focus on farmer-based programs, while potentially optimizing other surveillance strategies, is appropriate.

Thank you for this comment.

· For a paper that combines multiple pathogens, it would be beneficial to succinctly restate the different scales of outbreak sizes for ASF, PRRS, and APP seen in the simulations. This helps drive home the epidemiological significance of ASF in particular.

We have added the following sentences to the conclusion:

The simulation results revealed that ASF outbreaks were significantly larger in scale compared to PRRS and APP, highlighting the substantial threat ASF poses. To better prepare for its introduction in the Swiss swine industry, further research is needed.

Overall Recommendation

· The manuscript addresses an important and timely question about swine disease spread, leveraging comprehensive Swiss data.

· The modeling framework and results have broad relevance for designing optimal surveillance and early detection programs.

· While further clarity on parameter derivation and stronger emphasis on the limitations of tour data are needed, the study’s methodology, analyses, and conclusions are robust and compelling.

In summary, I recommend acceptance with minor to moderate revisions, focusing on:

· Enhanced clarity on the parameter choices and limitations (especially for indirect/fomite truck transmission).

· Additional organization/structuring in the Results section to aid readability.

· Emphasizing limitations associated with data gaps on transport tours and homogeneous mixing within farms.

With these adjustments, the manuscript would be a valuable contribution to the literature on swine infectious disease epidemiology and surveillance policy.

Reviewer #2

Thank you for recognizing me to review this manuscript. Generally speaking, this work is super excellent. I found the article so interesting and insightful, where all the sections, from the introduction to the conclusion, were well written and rich in content. It conveyed scientific information about the use of contact networks and mathematical modeling techniques in the identification of effective surveillance measures for swine pathogens. The authors have done a great job of reporting their findings to the scientific community regarding the effectiveness of surveillance measures in identifying the specific pathways of disease spread from one place to another. However, there are a few questions and suggestions I found attractive, and if properly addressed or cleared the doubt of the readers, then that would improve the work and reduce the ambiguity therein.
(A) Changes need to be made throughout the entire manuscript
i. The authors should be consistent in using either American or British type of spelling throughout the entire text, e.g., the spelling of a word modelling/modeling.

Thank you for this comment. We have changed the word modelling in the Introduction to modeling

Reviewer #3

The study is well-structured, methodologically rigorous, and addresses an important topic in veterinary epidemiology. The use of empirical Swiss transport data and stochastic modeling provides valuable insights into disease spread dynamics. The findings on farmer-based surveillance as the most effective strategy align with real-world observations (e.g., EFSA reports) and add practical relevance.

Key Recommendations for Revision
1. Data availability
The current statement explains restrictions due to third-party data (Identitas AG and FOAG). To comply with PLOS ONE's data policy, ensure the following:
Explicitly state the criteria for accessing data (e.g., contact information for Identitas AG and FOAG).
Confirm that data will be available indefinitely upon request.

Thank you for this comment. We would like to update our data availability statement. Here is the new statement to address your concerns:

Data cannot be shared publicly because of confidentiality concerns due to data belonging to third parties (the pig farmers themselves) and private companies. TVD and AGIS data are available from Identitas AG, Stauffacherstrasse 130A, 3014 Bern, Switzerland, https://www.identitas.ch/, and the Federal Office for Agriculture, 3001 Bern, Switzerland, https://www.blw.admin.ch/de/anwendung-agis, respectively and can be accessed for researchers who meet the criteria for access to confidential data. Requests are treated on an individual basis and signing a data sharing contract will be mandatory.

2. Figures and Tables
Figure Quality: Ensure high-resolution figures with axis labels, legends, and annotations.

We have ensured high resolution figures with axis labels, legends and annotations. Additionally, the images have all been converted to TIFF files.

For example:
Figure 1: Add a key explaining symbols (PRRS/APP cases, pig holdings).

Please see Figure 1 with the legend PRRS infection detected, APP infection detected and Pig holdings.

Figures 5–8: Clarify if medians/percentiles are shown for outbreaks or all simulations.

The captions for Figures 5-8 specify the source of the medians/percentiles. For example, the caption for Figure 5: Median, 95th percentile, and maximum cumulative infected number of farms for each disease without incorporating surveillance and a date of disease introduction in May, 2019. (A) All 1,000 simulation runs were included. (B) Only simulation runs with large outbreaks, as defined by 10 or more cumulative infected farms. Caption of Figure 6: Seasonal Changes. Median cumulative infected farms by date of pathogen introduction for simulations with large outbreaks. We have ensured that all figures specify if the data was based on outbreaks or all simulations.

Figure Citations: Reference figures in the correct order (e.g., Figure 3a/3b descriptions appear before Figure 4 in the text but are listed afterward).

Figure 3a/b appears on page 8 while Figure 4 appears on page 10

Table 1: Format consistently (e.g., superscripts for citations in the "Routes of Transmission" row).

The superscripts for citations in the Routes of Transmission row are all subscripts of a, b and c. We have ensured that they are all consistently formatted.

3. Clarity and language
Simplify complex sentences (e.g., in the Methods: "The stochasticity of the model is generated from the τ-leap method..." → "We used the τ-leap method with Poisson and binomial sampling to model stochasticity...").

Thank you for this comment. We have updated the manuscript with this text: *We used the τ -leap method with Poisson and binomial sampling to model stochasticity.*

Define all acronyms at first mention (e.g., SEIAR in the Abstract).

We do not use the acronym SEIAR in the Abstract. We use SIR which is defined as *susceptible-infectious-recovered (SIR).* When we first reference SEIAR in the introduction section we define it as indicated in the following sentence: *The within-herd disease dynamics are represented by a stochastic SEIAR (Susceptible, Exposed, Infectious, Asymptomatic, Removed/Recovered) model.*

4. Methodological Limitations
Tour data coverage: Emphasize that only 15% of transport tours were included and discuss how this might underestimate transmission via indirect contacts. Expand the sensitivity analysis discussion to highlight this limitation.

We state in the data description that “Since these two trader companies do not service all farms in Switzerland, we do not have the truck number, collection time, nor delivery time for all records of the TVD data.” We provide the exact coverage of 15%. All of section 2.10 is related to the shortcomings of the missing 85% and the sensitivity analysis that we had done. We also discuss this shortcoming in the Discussion section with an entire paragraph, starting with:
Due to the limited published estimates on transport tour transmission rates of direct and indirect truck sharing and truck fomites, setting the parameter values for our model was difficult…

Homogeneous mixing assumption: While acknowledged, briefly suggest how future work could incorporate heterogeneous mixing (e.g., farm size or housing systems).

In Section 2.3 Within-Farm Layer, we have updated the manuscript to provide rationale for our choice of homogeneous mixing with frequency dependent transmission. *In addition, we assumed homogeneous mixing of pigs at the farm with frequency dependent transmission. Although density-dependent transmission has often been used for animal diseases, this is more appropriate for animals kept in open spaces such on pasture where densities can change. Pig farms in Switzerland are strictly regulated with a clearly defined maximum number of animals per space by legislation. This leads to a maximum number of pigs per barn, limiting the number of contacts for each animal, even if the number of pigs per farm increases. Hence a frequency-dependent transmission assumption is more appropriate across farm systems in Switzerland.*

In addition, in the last paragraph of the Discussion section, we have updated the text which now states: *Expanding the model to capture the spread of ASF among wild boar, farm-specific population dynamics, nuanced seasonal changes with varying dates of introduction, the effect of additional tour data, and the outcome of ASF-specific control measures would provide further insight into the introduction and spread of ASF among Swiss swine. These improvements could refine the accuracy of model trajectories by incorporating more detailed information on disease reservoirs, transmission patterns, and the effectiveness of control measures.*

5. References
Ensure all in-text citations (e.g., "You et al., 2021") match the reference list.

We have confirmed that all in-text citations match the reference list. Here is the reference for "You et al., 2021": You, S., Liu, T., Zhang, M., Zhao, X., Dong, Y., Wu, B., Wang, Y., Li, J., Wei, X., & Shi, B. (2021). African swine fever outbreaks in China led to gross domestic product and economic losses. Nature Food, 2(10), 802–808.

Format references consistently (e.g., journal names in italics, proper use of "et al.").
We have confirmed that all references are consistent.

Minor Issues
Abstract: Specify the time frame for ASF detection ("8 weeks or more before") relative to other surveillance methods.

We have included the following sentence in the Abstract: Farmer-based surveillance programs were the only measures that consistently identified outbreaks of APP and PRRS and they were able to identify ASF outbreaks almost 8 weeks or more before active slaughterhouse- and network-based surveillance.

Results: In Figure 8, clarify whether error bars or confidence intervals are included for detection timelines.
Supplemental Material: Ensure all supplementary figures/tables are cited in the main text (e.g., "Suppl. Figure 3" is mentioned but not clearly linked to a specific result).

We have updated the caption of Figure 8 to include this sentence: *The boxplot shows the distribution of these dates, with the box representing the interquartile range (Q1 to Q3) and the line indicating the median.*

Journal Requirements:

When submitting your revision, we need you to address these additional requirements.

1. Please ensure that your manuscript meets PLOS ONE's style requirements, including those for file naming. The PLOS ONE style templates can be found at
https://journals.plos.org/plosone/s/file?id=wjVg/PLOSOne_formatting_sample_main_body.pdf and
https://journals.plos.org/plosone/s/file?id=ba62/PLOSOne_formatting_sample_title_authors_affiliations.pdf

The final version of the manuscript has been submitted using the PLOS LaTeX Template

2. Please note that PLOS ONE has specific guidelines on code sharing for submissions in which author-generated code underpins the findings in the manuscript. In these cases, we expect all author-generated code to be made available without restrictions upon publication of the work. Please review our guidelines at https://journals.plos.org/plosone/s/materials-and-software-sharing#loc-sharing-code and ensure that your code is shared in a way that follows best practice and facilitates reproducibility and reuse.

We ensure that the code will be shared as a public GitHub repository upon publication.

3. Thank you for stating the following in the Acknowledgments Section of your manuscript: This project was partially funded by the Swiss National Science Foundation (SNSF; project number 182404). Calculations were
performed on UBELIX (http://www.id.unibe.ch/hpc), the HPC cluster at the University of Bern. The data was provided by the
Identitas AG from the Federal Office of Agriculture (FOAG) and private pig traders.
We note that you have provided funding information that is not currently declared in your Funding Statement. However, funding information should not appear in the Acknowledgments section or other areas of your manuscript. We will only publish funding information present in the Funding Statement section of the online submission form.
Please remove any funding-related text from the manuscript and let us know how you would like to update your Funding Statement. Currently, your Funding Statement reads as follows:
SD, AC, and FG were supported by the Swiss National Science Foundation (SNSF:
https://www.snf.ch/en; project number 182404). The funders had no role in study
design, data collection and analysis, decision to publish, or preparation of the
manuscript.

Please include your amended statements within your cover letter; we will change the online submission form on your behalf.

We have removed the funding-related text from the manuscript. The Funding Statement should not change.

4. We note that you have indicated that there are restrictions to data sharing for this study. PLOS only allows data to be available upon request if there are legal or ethical restrictions on sharing data publicly. For more information on unacceptable data access restrictions, please see http://journals.plos.org/plosone/s/data-availability#loc-unacceptable-data-access-restrictions.

Before we proceed with your manuscript, please address the following prompts:

a) If there are ethical or legal restrictions on sharing a de-identified data set, please explain them in detail (e.g., data contain potentially identifying or sensitive patient information, data are owned by a third-party

organization, etc.) and who has imposed them (e.g., a Research Ethics Committee or Institutional Review Board, etc.). Please also provide contact information for a data access committee, ethics committee, or other institutional body to which data requests may be sent.

b) If there are no restrictions, please upload the minimal anonymized data set necessary to replicate your study findings to a stable, public repository and provide us with the relevant URLs, DOIs, or accession numbers. For a list of recommended repositories, please see https://journals.plos.org/plosone/s/recommended-repositories. You also have the option of uploading the data as Supporting Information files, but we would recommend depositing data directly to a data repository if possible.

We will update your Data Availability statement on your behalf to reflect the information you provide.

Please update the Data Availability statement to the following:

Data cannot be shared publicly because of confidentiality concerns due to data belonging to third parties (the pig farmers themselves) and private companies. TVD and AGIS data are available from Identitas AG, Stauffacherstrasse 130A, 3014 Bern, Switzerland, https://www.identitas.ch/, and the Federal Office for Agriculture, 3001 Bern, Switzerland, https://www.blw.admin.ch/de/anwendung-agis, respectively and can be accessed for researchers who meet the criteria for access to confidential data. Requests are treated on an individual basis and signing a data sharing contract will be mandatory.

5. We note that Figure 1 in your submission contain [map/satellite] images which may be copyrighted. All PLOS content is published under the Creative Commons Attribution License (CC BY 4.0), which means that the manuscript, images, and Supporting Information files will be freely available online, and any third party is permitted to access, download, copy, distribute, and use these materials in any way, even commercially, with proper attribution. For these reasons, we cannot publish previously copyrighted maps or satellite images created using proprietary data, such as Google software (Google Maps, Street View, and Earth). For more information, see our copyright guidelines: http://journals.plos.org/plosone/s/licenses-and-copyright.

We require you to either (1) present written permission from the copyright holder to publish these figures specifically under the CC BY 4.0 license, or (2) remove the figures from your submission:

a. You may seek permission from the original copyright holder of Figure 1 to publish the content specifically under the CC BY 4.0 license.

We recommend that you contact the original copyright holder with the Content Permission Form (http://journals.plos.org/plosone/s/file?id=7c09/content-permission-form.pdf) and the following text:
“I request permission for the open-access journal PLOS ONE to publish XXX under the Creative Commons Attribution License (CCAL) CC BY 4.0 (http://creativecommons.org/licenses/by/4.0/). Please be aware that this license allows unrestricted use and distribution, even commercially, by third parties. Please reply and provide explicit written permission to publish XXX under a CC BY license and complete the attached form.”

Please upload the completed Content Permission Form or other proof of granted permissions as an ""Other"" file with your submission.

In the figure caption of the copyrighted figure, please include the following text: “Reprinted from [ref] under a CC BY license, with permission from [name of publisher], original copyright [original copyright year].”

b. If you are unable to obtain permission from the original copyright holder to publish these figures under the CC BY 4.0 license or if the copyright holder’s requirements are incompatible with the CC BY 4.0

license, please either i) remove the figure or ii) supply a replacement figure that complies with the CC BY 4.0 license. Please check copyright information on all replacement figures and update the figure caption with source information. If applicable, please specify in the figure caption text when a figure is similar but not identical to the original image and is therefore for illustrative purposes only.
The following resources for replacing copyrighted map figures may be helpful:

USGS National Map Viewer (public domain): http://viewer.nationalmap.gov/viewer/
The Gateway to Astronaut Photography of Earth (public domain): http://eol.jsc.nasa.gov/sseop/clickmap/
Maps at the CIA (public domain): https://www.cia.gov/library/publications/the-world-factbook/index.html and https://www.cia.gov/library/publications/cia-maps-publications/index.html
NASA Earth Observatory (public domain): http://earthobservatory.nasa.gov/
Landsat: http://landsat.visibleearth.nasa.gov/
USGS EROS (Earth Resources Observatory and Science (EROS) Center) (public domain): http://eros.usgs.gov/#
Natural Earth (public domain): http://www.naturalearthdata.com/

We created Figure 1 ourselves using QGIS software. This is an open source software tool, and the image is not copyrighted.

6. Please include captions for your Supporting Information files at the end of your manuscript, and update any in-text citations to match accordingly. Please see our Supporting Information guidelines for more information: http://journals.plos.org/plosone/s/supporting-information.

We have renamed the Supporting information and included it at the end of the manuscript. We used the PLOS LaTeX template for the final manuscript submission.

7. Please review your reference list to ensure that it is complete and correct. If you have cited papers that have been retracted, please include the rationale for doing so in the manuscript text, or remove these references and replace them with relevant current references. Any changes to the reference list should be mentioned in the rebuttal letter that accompanies your revised manuscript. If you need to cite a retracted article, indicate the article's retracted status in the References list and also include a citation and full reference for the retraction notice.

We have reviewed the reference list and ensure that it is complete and correct.

Additional Editor Comments:

Please attend to the comments of the reviewers.

Please see all of the above responses.
