# Supplementary material for: Identifying effective surveillance measures for swine pathogens using contact networks and mathematical modeling
Source: PLoS One. 2025 Aug 22;20(8):e0329714. doi: 10.1371/journal.pone.0329714 (PMC12373290; doi:10.1371/journal.pone.0329714)
Supplement: Attachment — Submitted filename: Summary comments.docx [file pone.0329714.s015.docx]

**Manuscript title:** Identifying effective surveillance measures for swine pathogens using contact networks and mathematical modeling

**General Comments**

1. Relevance and Significance

- The manuscript explores a crucial topic, examining the spread of swine pathogens through multiple transmission pathways including direct transport, truck tours, and local spread and evaluating the effectiveness of different surveillance strategies in timely pathogen detection.
- The comparative approach using PRRS, ASF, and APP which have distinct biological and epidemiological profiles is particularly valuable. It highlights how differences in transmission modes affect the dynamics and detection of disease in pig populations.

2. Novelty

- The integration of three data sources (TVD records, pig trader data, and AGIS holdings data) to construct a complex, multi-layered contact network is a strong point of the study.
- Addressing both endemic (APP, PRRS) and emerging threats (ASF) in one modeling framework is quite innovative and provides a broader epidemiological scope than single-disease models.

3. Methodological Approach

- Overall, the approach (SEIAR within-herd model + dynamic, labeled, between-farm contact network) is logical and seems to capture key processes of disease transmission.
- The differentiation among direct transport, indirect truck-sharing, and fomites via truck drivers/equipment is valuable but still relatively underexplored in the literature. The authors make a genuine effort to incorporate real-world heterogeneities where data allow.

4. Clarity and Organization

- The manuscript is generally well-organized. The distinct sections (Introduction, Methods, Results, Discussion) are clearly delineated.
- Some sections, especially the Results, occasionally become quite dense when describing multiple pathogens and numerous surveillance scenarios. Additional structuring of the results possibly using subsections with descriptive headings would improve readability.

5. Limitations

- The study acknowledges data gaps on truck tours (only 15% coverage), which constrains definitive conclusions on indirect and fomite-based transmission. While the sensitivity analyses mitigate these concerns somewhat, the limitations should be emphasized more.
- The homogeneous mixing assumption at the farm level simplifies within-herd dynamics. Further consideration of farm size and housing system might affect the results, especially for highly transmissible pathogens (ASF) versus those with more variable clinical presentations (APP, PRRS).

6. Impact

- The findings on how quickly ASF can spread underscore the urgency for robust surveillance measures.
- The conclusions that farmer-based surveillance detects outbreaks earlier than other strategies has direct policy relevance particularly because it aligns with recent real-world experiences of ASF spread in Europe.
- The large potential for ASF spread in a naive country emphasizes the need for preparedness, which this model can inform.

**Section-by-Section Comments**

**Abstract**

1. The Abstract clearly summarizes the key objectives (assessment of contact types, evaluation of surveillance strategies).

2. You might emphasize the key quantitative finding: “Direct truck transport and local spread were found to be the main routes …” This is a strong conclusion that guides the reader upfront.

3. Consider briefly mentioning the major takeaway on surveillance (farmer-based strategies were the most effective).

**Author Summary**

1. The lay summary is concise and effectively highlights the real-world relevance.
2. The statement “It is not clear which contact type has the highest probability of transmission...” is appropriately answered in the subsequent text, but you might consider a more direct statement of your findings there.

**Introduction**

1. The motivation for comparing ASF, PRRS, and APP is clear, but you may want to highlight more systematically why these three were chosen as representative of distinct modes of transmission or clinical severity.
2. The reference to historical data (Figure 1) is helpful to show the Swiss context. Ensuring that the figure is easy to read and interpret is important. You could consider highlighting regions with prior cases more visibly (e.g., color-coding or labeling hotspots).
3. The hypotheses are generally well-articulated, but you might specify if you hypothesized that direct pig transports would be the most critical path or if you expected major differences in detection times among the three pathogens.

**Materials and Methods**

1. Data Sources (Section 2.2)

- The coverage of TVD, trader data, and AGIS is appropriate. The detail about not having the full sequence of truck stops for all transports is a key limitation. The authors do note this, but it merits repeated emphasis, given its impact on certain contacts (tour-based).
- It is beneficial that you combine farm attributes (farm type, pig numbers) with geographical coordinates. Clarify whether any missing data were excluded, or if imputation was performed for incomplete records.

2. Within-Herd Model (SEIAR) (Section 2.3)

- The compartmental structure is clearly described.
- The homogeneous mixing assumption is common but can lead to overestimation of outbreak size in large farms. A brief justification is provided; perhaps highlight any known empirical evidence that suggests frequency dependence within typical Swiss pig farms.

3. Between-Farm Contact Networks (Section 2.4)

- The categorization of edges (D, P, I, T, G) is thorough. The distinction among direct pig transport, shared truck contacts (direct/indirect), and truck fomites is valuable.
- For geographic edges, clarify why you selected a specific threshold \( k \) for distance (e.g., 5 km, 10 km). A sensitivity test for different distance cutoffs might strengthen the argument that “local spread” remains important even if the radius changes slightly.

4. Model Implementation and Parameters

- More detail on how the authors set the parameters for PRRS, ASF, and APP (especially for indirect truck sharing probabilities) would be useful. The references for these parameter estimates are critical for the credibility of the model.
- In the sensitivity analysis, the authors do a robust job exploring large multiplicative changes in tour-related parameters. This is well-executed but might benefit from additional clarity on how these factors relate to realistic upper/lower bounds from literature.

5. Surveillance Measures
   - The wide range of surveillance approaches (farmer-based with different participation and thresholds, slaughterhouse-based, targeted network-based) is helpful in illustrating detection trade-offs.
   - The frequency of sampling (every 90 days for targeted network-based) is an important assumption. Justify briefly why 90 days was chosen as opposed to, say, 60 or 120 days.

**Results**

1. Overall Disease Spread
   - The use of median, 95th percentile, and maximum outbreak sizes is appropriate, but it is easy for the reader to lose track of which pathogens are being referred to. Consider structuring the text so each pathogen is discussed in a separate subsection (or create tables/visual aids with side-by-side results).
   - Highlighting that only 16% of PRRS and 11% of APP simulations resulted in large outbreaks is an important detail. Possibly include a short explanation regarding whether that low probability is consistent with their known epidemiology in Switzerland.
2. Seasonality and Annual Trends
   - While you conclude no statistically significant differences in outbreak trajectories by introduction date, it might be helpful to present these results in simpler figures (boxplots or smaller sub-panels) for each pathogen. This would complement the discussion about the minor role of seasonality in Switzerland.
   - You reference a Kolmogorov–Smirnov test. Consider whether an additional measure like effect size or visual distribution plots might clarify these comparisons.
3. Routes of Transmission
   - The percentage breakdown of between-farm spread is a key output. Showing direct pig transfers and local spread as major drivers is a critical conclusion. The minimal role of tours (<1% on average) is surprising yet informative.
   - Ensure you clarify that these results hinge on partial data for tours. Emphasize that the sensitivity analyses indicate that large multiplication of tour parameters does not drastically change the outcome (except a slight bump for APP).
4. Surveillance Findings
   - The detection-time comparisons are crucial for policy. The results, especially regarding the early detection by farmer-based strategies for ASF, are compelling. It would help to provide a single table summarizing average detection time (in days) and the average cumulative infected farms/pigs across all surveillance strategies for each pathogen.
   - The differences in performance between slaughter-based and farmer-based strategies could be contextualized in an overall cost or feasibility discussion (though a formal cost–benefit analysis is suggested for future work).

**Discussion**

1. Interpretation of Main Findings
   - The discussion does a good job contextualizing the results in the broader literature. The strong conclusions regarding the dominance of direct pig transport and local spread, as well as the effectiveness of farmer-based surveillance, are well-articulated.
   - The authors might consider discussing whether geographic spread includes airborne transmission or primarily anthropogenic factors (veterinary visits, shared equipment, etc.). Clarify if future work could stratify or parse those different local routes.
2. Model Limitations and Future Directions
   - The limitations (partial truck tour data, homogeneous mixing, frequency dependence) are addressed, but consider emphasizing that real-world data on contact intensity, farm biosecurity, and pig flow patterns might refine these estimates.
   - Future directions such as including wild boar transmission for ASF, or incorporating more realistic within-farm population structures, are important. Expanding the discussion on how these improvements might change the key outcomes (especially for ASF) would be helpful.
3. Policy Implications
   - The conclusion that farmer-based surveillance detects outbreaks faster is consistent with the recently observed ASF outbreaks in Europe and with EFSA’s conclusions. Emphasizing how to encourage or mandate better on-farm reporting could strengthen the policy relevance.
   - The manuscript might benefit from additional commentary on how these findings could be translated into Swiss or EU-level regulations to enhance early disease detection.
4. Conclusions
   - The final paragraph or “Conclusion” section effectively reiterates the main take-home messages. The recommendation to focus on farmer-based programs, while potentially optimizing other surveillance strategies, is appropriate.
   - For a paper that combines multiple pathogens, it would be beneficial to succinctly restate the different scales of outbreak sizes for ASF, PRRS, and APP seen in the simulations. This helps drive home the epidemiological significance of ASF in particular.

**Overall Recommendation**

- The manuscript addresses an important and timely question about swine disease spread, leveraging comprehensive Swiss data.
- The modeling framework and results have broad relevance for designing optimal surveillance and early detection programs.
- While further clarity on parameter derivation and stronger emphasis on the limitations of tour data are needed, the study’s methodology, analyses, and conclusions are robust and compelling.

In summary, I recommend acceptance with minor to moderate revisions, focusing on:

- Enhanced clarity on the parameter choices and limitations (especially for indirect/fomite truck transmission).
- Additional organization/structuring in the Results section to aid readability.
- Emphasizing limitations associated with data gaps on transport tours and homogeneous mixing within farms.

With these adjustments, the manuscript would be a valuable contribution to the literature on swine infectious disease epidemiology and surveillance policy.

Dr. Mohamed Bakheet

Feb 13, 2024
